# Bioavailability of Phenolic Compounds in Californian-Style Table Olives with Tunisian Aqueous Olive Leaf Extracts

**DOI:** 10.3390/molecules28020707

**Published:** 2023-01-10

**Authors:** Dalel Mechi, Bechir Baccouri, Daniel Martín-Vertedor, Leila Abaza

**Affiliations:** 1 Laboratory of Olive Biotechnology, Centre of Biotechnology of Borj-Cedria (CBBC), Hammam-Lif 2050, Tunisia; 2 Department of Biology, The Faculty of Science of Bizerte, University of Carthage, Zarzouna 7021, Tunisia; 3 Technological Institute of Food and Agriculture (CICYTEX-INTAEX), Junta of Extremadura, 06007 Badajoz, Spain

**Keywords:** olive leaf extract, biodisponibility, phenol profile, olives, Tunisian

## Abstract

Recent advances in biotechnology have ensured that one of the main olive tree by-products is olive leaf extract (OLE), a rich source in bioactive compounds. The aim of this work was to study the phenolic composition in different OLEs of three Tunisian varieties, namely, ‘Sayali’, ‘Tkobri’, and ‘Neb Jmel’. The in vitro biodigestibility effect after ‘Sayali’ OLE addition to Californian-style ‘Hojiblanca’ table olives was also studied. This OLE contained bioactive molecules such as hydroxytyrosol, tyrosol, oleropeine, Procianidine B1 (PB1), and *p*-cumaric acid. These compounds were also found in fresh olives after OLE was added. Furthermore, from fresh extract to oral digestion, the detected amount of bioavailable phenol was higher; however, its content decreased according to each phase of gastric and intestinal digestion. In the final digestion phase, the number of phenols found was lower than that of fresh olives. In addition, the phenolic content of Californian-style ‘Hojiblanca’ table olives decreased during the in vitro digestion process. The antioxidant activity of this variety decreased by 64% and 88% after gastrointestinal digestion, being the highest antioxidant capacity found in both simulated gastric and intestinal fluid, respectively. The results show us that the ‘Sayali’ variety is rich in phenolic compounds that are bioavailable after digestion, which could be used at an industrial level due to the related health benefits.

## 1. Introduction

The olive tree (*Olea europaea*, Oleaceae) has important economic, social, and cultural value within the Mediterranean area. This species is mainly used to market virgin olive oil and table olives globally [1,2]. In addition, 10% of the by-products generated by these industries, such as olives twigs and leaves, have no practical applications. During the pruning of these trees by farmers, olive leaves are produced as a by-product without any added value [3]. In recent years, different research has found new strategies to revalorize these by-products of olive industries, managing to obtain added value from the product, not only for an environmental objective but also for the healthy properties of these by-products [4].

Olive leaves have been used for many centuries in traditional medicine against several diseases. The beneficial properties of the leaves’ preparations are mostly attributed to their richness in polyphenols, which are numerous and of a diverse nature [2,5,6]. In fact, a lot of research has revealed that the phenolic compounds of olive leaves are important ingredients, which provide enormous health benefits for humans [7]. A large variety of pharmaceutical studies have shown that olive leaf extract presents health properties with its antioxidant, anti-mutagenicity, anti-cancer, anti-carcinogenesis, anti-microbial, anti-diabetes, and anti-inflammation activity [8,9,10,11].

The phenolic compounds of olive leaves are classified as secondary metabolites that efficiently inhibit reactive oxygen species. This product shows bioactive compounds able to prevent oxidative stress against free radicals’ products in the body such as reducing agents or being donators of hydrogen or metal chelators [4,12]. Up until now, research has taken a special interest in the natural antioxidant compounds. Such a by-product has the advantage of being available throughout the entire olive season and is a great and cheap source of phenolic compounds [13,14].

Solvent extraction is a way to extract the phenolic compounds present in this natural matrix [13]. However, several researchers have studied the efficiency of phenol extraction, indicating that it depends on factors such as the type, proportion, and temperature of the solvent used [15]. In this sense, the types of the extraction and solvent could two of the most relevant factors to optimize in order to achieve a high content of healthy bioactive compounds. [16]. As a matter of fact, there is a paucity of information regarding the estimation of the most effective solvent for the extraction of antioxidant components from a target plant [17].

Nowadays, the identification of bioactive compounds from Tunisian olive leaf varieties has gained an international focus. In this context, Abaza et al. [18] indicated that some phenol extractions from the Tunisian ‘Gerboui’ variety such as apigenin 7-glucoside contribute to leukemia HL-60 cells in humans. Furthermore, for many occasions, it is more interesting to obtain complex phenolic extracts from plants than isolate specific compounds due to the synergistic effect of these more complex phenolic matrices [19].

Spanish-style table olives, among fermented foods, are considered one of the most popular products in the world due to their sensory properties and their high content of bioactive compounds, such as phenols, monounsaturated fatty acids, vitamins, and minerals [20]. The ‘Hojiblanca’ variety is the main table olive in the Mediterranean region in Spain. Furthermore, Californian-style black olives also require a popular elaboration process, for which there are many industries around the world. It creates a shiny black colour that is highly attractive to consumers [21]. This elaboration process consists of harvested green olives being submitted to a lye treatment to remove bitterness, while an oxidation process with the air occurs. Finally, the darkened olives are treated with an iron salt and subjected to a thermal sterilization process to microbiologically stabilize the product [22].

Understanding the mechanisms of release of olive leaf extract (OLE) and table olive compounds, especially the ones considered beneficial for human health, is crucial to keep up this research. To reach that goal, the bibliography contains different digestion models that artificially simulate the gastrointestinal tract [23]. With these methods, it is possible to verify the bioavailability and effectiveness during their function explication. In vivo methods, which are more accurate and effective, are discounted by their slowness and expensiveness. Fortunately, in vitro methods are quicker and cheaper but do not have the same accuracy than in vivo procedures, due to the complexity of any natural method. In order to check if in vitro methods are suitable for a scientific study and the levels of information it provides, different researchers have studied different in vitro gastrointestinal digestion models to assess the bioavailability of the bioactive chemical compounds in foods [24]. The main conclusion gives us an idea of the bioavailability of certain compounds after being subjected to gastrointestinal processes, despite certain real differences between the both methods.

The present work aims to describe the optimization of olive leaves’ fractionation for different Tunisian varieties, which have not been clearly investigated, using solvents with increasing polarity and water. A fast screening and comparison between the studied varieties was completed, in an attempt to establish the best cultivar in terms of its phenolic compounds content and antioxidant capacity. Then, to investigate the bioavailability of OLE and table olives of the ‘Hojiblanca’ variety, a biochemical in vitro digestion was applied.

## 2. Results and Discussion

### 2.1. Phenolic Compounds in OLE

The phenol profiles of the OLE from different Tunisian varieties are shown in Table 1. Significant quantitative differences were presented in certain phenols in the Tunisian varieties that were analysed. Thus, it was found that bioactive molecules in OLE such as hydroxytyrosol, oleuropein, and luteolin-7-*O*-glucoside are the most abundant phenolic compounds in all the studied varieties, followed by verbascoside, quercetin, quercetin-3-rutinoside, caffeic acid, chlorogenic acid, epicatequine, vainillic acid, gallic acid, and *p*-cumaric acid. In fact, oleuropein, hydroxytyrosol, and luteolin-7-*O*-glucoside represented more than 90% of the total identified compounds. Other researchers also quantified the phenol profile in other OLEs, finding phenols such as oleuropein, hydroxytyrosol, and luteolin-7-*O*-glucoside [19,25,26].

The OLE fractions (hexane, diethyl ether, ethyl acetate, and methanol) and aqueous extracts presented high polyphenol content, despite a significant variability that occurred, depending on solvent polarity and variety (Table 1). These results confirmed that OLE is a good source of phenolic compounds. As presented in Table 1, the highest values were registered in the methanol fraction for all the studied varieties, closely followed by ethyl acetate and diethyl ether fraction, whereas hexane exhibited the lowest phenolic contents. Mechi et al. [5] also found a high phenol concentration in the ‘Chetoui’ olive variety with a methanol solvent. The maximum concentrations were found for oleuropein, hydroxytyrosol, and luteolin-7-*O*-glucoside (275,059, 45,324, and 15,642 mg 100 g^−1^, respectively) in ‘Sayali’ sample extracted with methanol. Therefore, the solvent used to extract phenols resulted in being a significant factor regarding the amount of the total phenols’ concentration obtained (*p* < 0.05). The methanol and ethyl acetate solvents extracted phenols from olive leaves in a high concentration due to their lower solubilities and polarities. Thus, these polar solvents were more effective at extracting phenols than the rest of those that were used [27,28]. Also of note is that the aqueous extraction presented a high content of phenols, an interesting aspect regarding the use of these by-products by the agri-food industry. Many previous reports confirmed these results. Medini et al. [29] found similar results when using several solvents with increasing polarity (hexane, dichloromethane, ethanol, and methanol) in the splitting of the medicinal halophyte, *Limonium densiflorum*. According to these authors, the methanol fraction had the highest phenolic compounds, followed by the ethanol, dichloromethane, and hexane fractions.

Consequently, obtaining polyphenols from plant matrices is directly related to the solubility, type, and polymerization and the interaction of phenols with the solvent used for extraction [16]. Therefore, the choice of phenolic extraction method for a plant material is a complex choice, since those with a lower polarity are generally considered to have more extracting power for lipophilic compounds, such as aromatic compounds [30]. Meanwhile, polar solvents are used for hydrophilic phenols, such as carbohydrates, glycosides, and amino acids [31]. Moreover, the phenolic compounds’ quantification showed a large variability depending on the variety. The varieties that presented the highest concentration of phenols were ‘Sayali’ followed by ‘Tkobri’, while ‘Neb Jmel’ presented a lower amount. Generally, ‘Sayali’ was found to be the variety with the highest phenol profile (Table 1).

**Figure 1:**
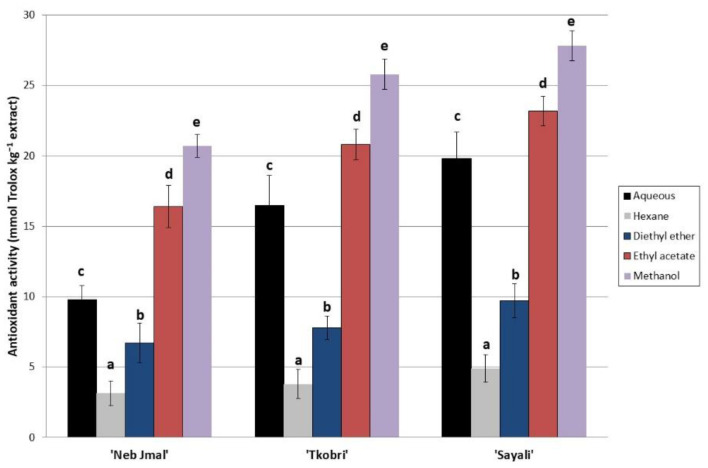
Antioxidant activity of OLE from ‘Sayeli’, ‘Tkobri’, and ‘Neb Jmel’ varieties extracted with different organic and aqueous solvents. Lowercase letters (**a**–**e**) mean statistically significant differences between different solvents used for each variety (Tukey’s HSD test, *p* < 0.05).

The antioxidant activity was also assessed, and the results are in line with those found for the phenolic profile (Figure 1). The highest antioxidant activity of the OLE from the Tunisian varieties corresponded to the methanol and ethyl acetate extracts from the OLE of the ‘Sayali’ variety. Significant differences were found between both the solvents and varieties used. The solvent used in this study with the least antioxidant activity was hexane. Similar results were also found with the ‘Chetoui’ olive variety by Mechi et al. [5]. These authors confirmed the high antioxidant activity of other local Tunisian varieties. The antioxidant activities of the adult and young ‘Chetoui’ variety were less than those found in our study with the ‘Sayali’ variety. Thus, the antioxidant properties of the OLEs from the different varieties studied such as the ‘Arbequina’ [32], adult ‘Chetoui’ [5], and ‘Chemlali’ [33] varieties presented values of 15.6, 18.5, and 19.1 mmol Trolox kg^−1^ of the extracts, respectively. We have to highlight that these OLEs from the Tunisian varieties presented a 15-fold higher amount of antioxidant activity than those obtained from the virgin olive oil of the ‘Picual’ variety [32]. Finally, the OLEs from the ‘Oleaster’, ‘Chaaibi’, and ‘Zarrazi’ varieties presented antioxidant activity values of 12, 10, and 8 mmol Trolox kg^−1^ of OLE, respectively [6].

### 2.2. Phenolic Compounds Effect of ‘Sayali’ Aqueous Extract after In Vitro Gastrointestinal Evaluation

The individual phenolic compounds were analysed to characterize the aqueous OLE after in vitro gastrointestinal digestion. This extract may be very useful, since it is easily obtained and showed feasibility for industrial production. It is important to note that solvent extracts were rejected owing to their toxicity [32], so water was used as extractant instead. As shown in Table 2, the number of phenols showed significant differences (*p* < 0.05) among the different digestion phases.

The gastrointestinal digestion process was evaluated by quantifying the phenols in each of the aqueous phases of the process: the oral (O), gastric (G), small intestine (SI), and large intestine (LI) phases. Furthermore, these results were also compared with the phenols obtained for the digestion process without the addition of any microorganisms in the large intestine, to find out the effect of such microorganisms on the phenol composition present in OLE. In the O phase, the phenolic compounds’ bioavailability was the highest. The chemical and enzymatic actions in the O phase were enough to release these compounds from the matrix (Table 2). Afterwards, the bioavailability of the phenols decreased in the ensuing gastrointestinal digestion with the degradation of the OLE matrix, reaching the minimum value in LI phase.

The initial amounts of these compounds in OLE were slightly released after being submitted to in vitro G digestion. Therefore, the phenols would be released to enter into the intestinal tract. In the SI phase, the phenolic compounds’ bioavailability kept decreasing (Table 2). Finally, portions of the phenols released were obtained in the LI phase. Therefore, a large number of phenols was lost by excretion. The lowest values that were found corresponded to the LI digestion phase, with respect to the fresh extract. Finally, despite the fact that the OLE is an easy matrix to be degraded during digestion, the phenol composition present in the OLE matrix after gastric and duodenal digestion was very significant, especially hydroxytyrosol, oleuropein, and tyrosol, which are some of the most abundant phenols in olives.

In addition, with the purpose of understanding if there is any microbial activity, a digestion without microorganisms was completed. As we can see in Table 2, there were significant differences between the amount of phenolic compound bioavailability in the large intestine compared with the control large intestine (C-LI) phase. The lowest values were found in the digestion phase, which was degraded by the action of the inoculated microorganism. Thus, the phenolic compound reduction ranged between 8% and 20%. The microbial effect on the phenolic content is discussed in Section 2.3.

### 2.3. Influence of Aqueous Extracts in Californian-Style Black Olives

#### 2.3.1. Phenol and Antioxidant Activity of ‘Hojiblanca’ Table Olives after OLE Addition

The Table 3 presents the phenolic composition of the ‘Hojiblanca’ variety which was elaborated by the addition of ‘Sayali’ OLE extract. The major phenols found were oleuropein and hydroxytyrosol (HTy). The maximum concentration of the phenolic compounds was 3460.9 mg 100 g^−1^ for oleuropein, followed by 2175.5 mg 100 g^−1^ for HTy, with an OLE addition at a 1:10 ratio. There is abundant literature supporting this observation [34,35,36]. The levels of the phenolic compounds decreased significantly after the Californian-style thermal sterilization process, while quercetin, caffeic acid, and chlorogenic acid did not show significant content. On the other hand, no significant differences were observed in the content of epicatechin, verbascoside, vanillic acid, quercetin-3-rutinoside, and luteolin-7-*O*-glucoside. Phenolic compounds are thermosensitive and easily degrade at high temperatures [32]. However, when the addition of OLE at a 1:10 ratio was completed for table olives, an increase of 31.4 times for oleuropein, 4.67 times for hydroxytyrosol, and 237.5 times for luteolin-7-*O*-glucoside can be seen. Vanillic acid was the only one that did not demonstrate a concentration variation. Various researchers verified that the use of an OLE addition in other foods improves the bioactive content present in these types of olives that were previously sterilized, so implementation at an industrial level should be considered, since it is an economical and a rich source of phenols that could be used as a food additive [35,37].

**Figure 2:**
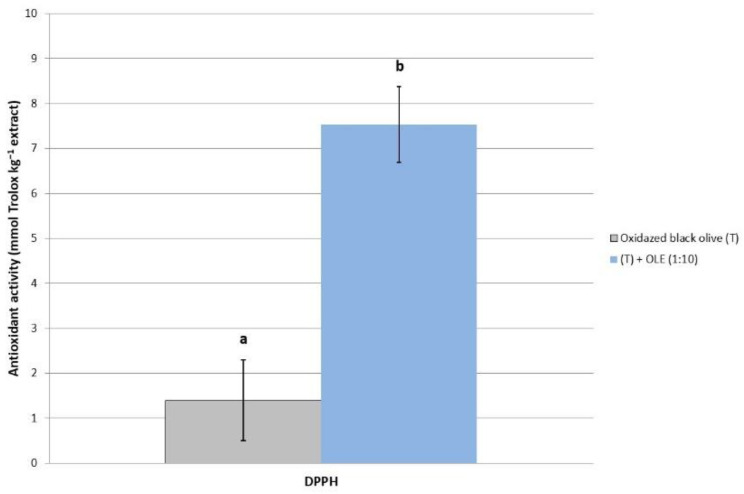
Antioxidant activity of ‘Hojiblanca’ variety with the addition of ‘Sayali’ aqueous extract. Lowercase letters (**a**, **b**) mean statistically significant differences (Tukey’s HSD test, *p* < 0.05) among table olives after OLE addition. DPPH: 1,1-diphenyl-2-picrylhydrazyl.

Regarding the antioxidant activity of the (oxidized black olive (T)) + OLE (1:10) treatment, which is tested herein (roughly 7.5 mg Trolox g extract^−1^), it is clearly higher compared to the ‘Hojiblanca’ table olive without an OLE addition (Figure 2). In addition, when compared with the studied samples, the (T) + OLE (1:10) treatment had five-fold higher antioxidant activity than the ‘Hojiblanca’ table olive without an OLE addition. Interestingly, the OLE of the ‘Sayali’ variety presented a high antioxidant capacity, which may be due to the high amounts of hydroxityrosol, tyrosol, and, especially, oleuropein that were found. Thus, the OLE addition of this variety to Californian-style black olives also provoked a high antioxidant activity for the table olives [38,39]. These results would be useful in relevant industries, since the design of functional foods should provide extra added value, because the bioactive compounds would indirectly serve to improve the risk of suffering from ageing-related diseases and to reduce oxidative stress in humans [40].

#### 2.3.2. Gastrointestinal Activity of Californian-Style ‘Hojiblanca’ Table Olives after OLE Addition

As expected, after in vitro digestion evaluation, the phenolic profile of the table olives with an OLE addition of the ‘Sayali’ aqueous extract showed significant differences (*p* < 0.05) (Table 4). The phenols of the table olives with the ‘Sayali’ aqueous extract presented a high concentration after oral administration (O phase). Afterwards, the number of phenols decreased during each step of the digestion phases. In the SI phase, the release of these compounds kept decreasing, caused by the acid digestion of the following digestion phases. Finally, it should be noted that, although in the LI phase the number of phenols was present in a low concentration, this is an interesting aspect, since these released phenols could be absorbed into the organism. In this sense, the most relevant phenols present after being subjected to the different phases of digestion were hydroxytyrosol, oleuropein, luteolin-7-*O*-glucoside, verbascoside, quercetin, and quercetin-3-rutinoside. Thus, the ‘matrix effect’ proposed by Lodolini et al. [41] could explain that a large number of phenols were released in the final phase of the digestion of the olives with an OLE addition. The matrix complexity of the olive protects from phenol degradation during the gastrointestinal digestion process. On the contrary, the digestion of simple matrices, such as aqueous extracts [23,37], provokes the phenols that decreased in the last part of the gastrointestinal process.

The effect of the intestinal microbiota on the content of the phenolic compounds in olives with an OLE addition was also studied (Table 4). Differences were observed between the phenols’ bioavailability in the olives in the LI phase compared with the C-LI. The lowest values were observed in the LI phase; the phenol concentration was reduced by 11–12%. The chemical activity was not able to release phenols from the complex olive matrix (Table 4). In addition, the bioavailability decreased when the matrix was degrading in the ensuing stages of gastrointestinal digestion, which showed the lowest values in the LI.

These results are interesting, since the literature indicates that most bioactive compounds are absorbed during an advanced stage of digestion [42]. These results are in agreement with those found by Liu [43], with further evidence found by epidemiological studies [44]. Thus, microbial activity is crucial in gastrointestinal digestion (Table 4). These results show us that microorganisms can degrade phenols after the metabolism that occurs in intestinal digestion by the bacterial fermentation process [37]. In fact, different researchers [41,45,46] have indicated that phenols’ structure can be changed by different chemical degradations such as ring opening, methylation, and ring reductions. Other researchers studied the effect of microbial action on flavanols in the colon [45]. The enzymatic actions present in humans, such as cinnamoyl esterase, have microbial origins [47]. Researchers also indicated the benefit of releasing bioactive compounds during the last phase of the digestive process, especially to reduce oxidative damage in erythrocytes [46]. The results obtained are promising, since an OLE addition to table olives would cause a substantial increase in the bioactive compounds that would be more bioavailable in the human body; these results would have to be confirmed with others using in vivo assays. Thus, when free phenols are absorbed during gastrointestinal digestion, they could contribute to a high antioxidant action, when in vivo models are studied [48].

## 3. Materials and Methods

### 3.1. Reagents and Standards

Hexane, diethyl-ether, ethyl acetate, and methanol solvents used for phenol extraction were purchased from Sigma-Aldrich Chemie (Steinheim, Germany). For the elaboration of Californian-style black table olives, acetic acid (Panreac Applichem, Darmstadt, Germany), salt, sodium chloride, ferrous gluconate (Sigma-Aldrich, St. Louis, MO, USA), sodium hydroxide (Sigma-Aldrich, St. Louis, MO, USA), and calcium chloride (Tetra Chemicals, Helsingborg, Sweden) were used. For phenol analysis, different standards of hydroxytyrosol, procyanidin B1 (PB1), verbascoside, and oleuropein were supplied by Extrasynthése (Genay, France) and Quercetin by MERK-Schuchardt (Hohenbrunn, Germany). Tyrosol, epicatechin, vanillic acid, gallic acid, quercetin-3-rutinoside, caffeic acid, chlorogenic acid, and luteolin-7-*O*-glucoside were purchased from Sigma-Aldrich Chemie (Steinheim, Germany), and *p*-coumaric acid and were supplied by FlukaChemie (Steinheim, Germany). For the preparation of mobiles phases, high-performance liquid chromatography (HPLC)-grade methanol and acetonitrile were purchased from Fisher (Loughborough, UK), and P.A.-grade formic acid was obtained from PANREAC (Barcelona, Spain). Sodium fluoride was purchased by Sigma-Aldrich Chemie (Steinheim, Germany). For antioxidant activity, 1,1-diphenyl-2-picrylhydrazyl (MERK-Schuchardt, Hohenbrunn, Germany), methanol, and Trolox (Sigma-Aldrich Chemie, Steinheim, Germany) were purchased. For digestion gastric fluid, pepsin (MERK-Schuchardt, Hohenbrunn, Germany) and sodium chloride (Sigma-Aldrich Chemie, Steinheim, Germany) were obtained. For intestinal fluid, pancreatin (Sigma-Aldrich Chemie, Steinheim, Germany), lipase (MERK-Schuchardt, Hohenbrunn, Germany), cholic, and deoxycholic acids (Sigma-Aldrich Chemie, Steinheim, Germany) were purchased. The large intestinal phase consists of inoculation with *Lactobacillus* and *Escherichia coli*, which were purchased from Sigma-Aldrich Chemie (Steinheim, Germany).

### 3.2. Plant Material

The olive leaves of three different olive tree varieties, namely, ‘Sayali’, ‘Tkobri’, and ‘Neb Jmel’, were collected from the region of Takelsa, Nbeul (in the northeastern part of Tunisia) during the maturing fruit season. The raw material was washed, dried, and ground at room temperature. Furthermore, olives (*Olea europaea* L.) of ‘Hojiblanca’ variety were obtained from olive groves located in the ‘Vegas Bajas del Guadiana’ region. In total, 100 kg of ‘Hojiblanca’ variety olives were harvested.

### 3.3. Extraction of Bioactive Compounds

The extraction of bioactive compounds from olive leaves was done using different solvents (hexane, diethyl-ether, ethyl acetate, and methanol) with different polarities [5]. Furthermore, aqueous extract was obtained following the methodology proposed by Delgado-Adámez [32].

### 3.4. Californian-Style Black Olives’ Elaboration Process

Olives of ‘Hojiblanca’ variety were processed following the methodology described by Martín-Vertedor et al. [21] for Californian-style black olive. Olives were stored in brine solution for 4 months, and after that they were submitted to lye treatment and oxidation process. Olives were neutralized at pH 7, and ferrous gluconate was added to fix the black colour. Finally, olives were introduced into cans with the corresponding additives [21].

### 3.5. HPLC Analysis of Phenolic Compounds

For polyphenols extraction, the methodology described by Cabrera-Bañegil et al. [49] was used. The extract was centrifugated for 10 min at 4 °C at 10,000 rpm. After the supernatant was filtered, it was injected into an Agilent 1100 model HPLC system (Hewlett-Packard, Waldbronn, Germany). The detectors used were diode array (DAD) and fluorescence detector (FLD) for the identification of phenol profile. All the identified compounds were quantified by using phenol standards. For chromatographic identification of the phenol being studied, a Gemini-NX C18 column (150 × 4.6 mm i.d., 3 µm thickness, Phenomenex) was used. The mobile phases were composed of formic acid (0.1%) in aqueous (A) and acetonitrile (B) phases. The following gradient system was supplied: 0–1 min, 3% B; 1–30 min, linear gradient from 3% to 35% B; 30–33 min, from 35% to 50% B; 33–34 min, from 50% to 100%; 34–50 min, 100% B isocratic. Then, 10 μL of the sample was injected in the HPLC, and the flow rate was 1 mL min^−1^ at 40 °C. DAD detector was used for determination of gallic acid at 280 nm; quercetin, oleuropein, and *p*-coumaric acid at 255 nm; verbascoside and chlorogenic acid at 320 nm; and apigenin, luteolin, and their correspondent glucosides forms at 350 nm, while FLD at 275/315 nm allowed the quantification of hydroxytyrosol, tyrosol, PB1, epicatechin, and vanillic acid.

### 3.6. Antioxidant Activity

The antioxidant activity was analysed in the different samples using the 1,1-diphenyl-2-picrylhydrazyl (DDPH) method [5]. In total, 300 µL of the samples were introduced into 2.7 mL of 1,1-diphenyl-2-picrylhydrazyl (DPPH), and the mixture was kept in the dark for 1 h. Afterwards, the samples were read by UV-3100 Spectrophotometer (Selecta, Barcelona, Spain) at 517 nm. A blank sample was made with Methanol (J.P. Selecta, S.A) and analysed with the spectrophotometer. The results were expressed in mmol Trolox kg^−1^ extract in Trolox equivalent antioxidant capacity (TEAC).

### 3.7. Simulated Gastrointestinal Digestion

The following method was used for both aqueous OLE and table olives with OLE addition. To study the behaviour of phenols during the different digestion phases, OLE from ‘Sayali’ and Californian-style ‘Hojiblanca’ table olives were submitted to an in vitro assay [46,50]. Gastrointestinal digestion consisted of digesting the sample in the different phases: oral, gastric, and intestinal. First, 0.5 g of samples was exposed and mixed with 1 mL of natural human saliva. The mix was stored in an orbital shaker incubator (Optic ivymon system) for 10 s at 37 °C. This aqueous liquid mix was incubated by orbital shaking with 3.6 mL of simulated gastric fluid that contained pepsin and sodium chloride at pH 2.2 for 20 min at 37 °C. After that, the liquid was submitted to a small intestine digestion by exposure to 3.6 mL of simulated intestinal fluid that contained pancreatin, lipase, cholic, and deoxycholic acids in a phosphate-buffered saline (PBS) buffer. The mixture obtained was stirred for 20 min at 37 °C to complete the intestinal digestion. To verify the microbial effect on the samples during process, a digestion was performed in the large intestine by inoculating 10^5^ colony-forming unit (CFU) of *Lactobacillus* and *Escherichia coli* per mL. The mix was incubated at 37 °C by orbital shaking for 4 h. Finally, after each digestion step, the mixture was centrifuged at 21,036× *g* at 4 °C for 10 min to remove solid particles. The supernatant was filtered and then injected into the HPLC system. All the assays were done in triplicate to quantify phenol composition.

### 3.8. Statistical Software

SPSS 17.0 software was used (SPSS Inc. Chicago, IL, USA) to perform the statistical analysis. Data were expressed as mean values followed by the standard deviation (SD) and were analysed using a one-way analysis of variance (ANOVA), followed by Duncan’s multiple range test. The significance level was set at *p* < 0.05.

## 4. Conclusions

The highest concentrations of phenolic compounds that were obtained from olive leaves were by using the methanol and ethyl acetate solvents; however, the aqueous extraction presented a significant content of phenols that allowed for having them in a natural aqueous matrix suitable to be used in the agri-food industry. These phenols are useful for discriminating among the evaluated varieties. The variety with the highest phenolic compounds was ‘Sayali’, followed by ‘Tkobri’. The ‘matrix effect’ can justify the digestion-phases tendency, which tells us that complex matrixes such as the Californian-style table olive could protect the phenols to be degraded by gastric acids or microbial attack. Thus, such compounds are going to be bioavailable in the final steps of digestion, to be absorbed by the human body. For that reason, simple matrixes may be not beneficial to assimilate a high quantity of phenolic compounds. In addition, in the final phase of digestion, it was observed that microbial activity caused a decrease in the phenolic profile of the studied samples. This aspect suggests the need to use complex matrices during the digestion phases that help to protect phenols from gastrointestinal degradation.

## Figures and Tables

**Table 1:** Polyphenols’ concentration of olive leaf extract (OLE) extracted by different solvents and aqueous extracts from different varieties (mg 100 g^−1^).

**Variety**	**E**	**Hydroxytyrosol**	**Tyrosol**	**PB1**	**Epicatequin**	**Verbascoside**	**Quercetin-3-rutinoside**	**Luteolin-7-*O*-glucoside**
‘Sayali’	1	9278.0 ± 14.4 ^c^	258.8 ± 6.3 ^b^	120.5 ± 7.9 ^b^	115.3 ± 0.03 ^c^	782.7 ± 12.9 ^c^	709.4 ± 3.8 ^b^	2007.9 ± 7.3 ^b^
	2	325.9 ± 14.3 ^a^	15.9 ± 1.4 ^a^	12.9 ± 2.4 ^a^	8.8 ± 2.6 ^a^	45.1 ± 1.8 ^a^	45.5 ± 2.4 ^a^	120.4 ± 10.4 ^a^
	3	3970.1 ± 33.85 ^b^	292.1 ± 0.8 ^c^	115.3 ± 5.6 ^b^	119.8 ± 2.6 ^c^	648.6 ± 14.7 ^b^	689.7 ± 6.5 ^b^	1983.6 ± 6.8 ^b^
	4	15,841.1 ± 46.0 ^d^	306.5 ± 2.9 ^c^	189.6 ± 9.9 ^c^	130.9 ± 2.6 ^d^	850.6 ± 11.0 ^d^	864.7 ± 8.9 ^c^	2213.5 ± 21.4 ^c^
	5	45,324.0 ± 119.1 ^e^	480.0 ± 1.3 ^d^	212.6 ± 10.2 ^d^	109.9 ± 5.8 ^b^	1236.8 ± 14.0 ^e^	1224.2 ± 24.8 ^d^	15,642.8 ± 444.8 ^d^
‘Tkobri’	1	7589.6 ± 85.8 ^c^	168.6 ± 5.2 ^c^	104.7 ± 1.6 ^b^	42.7 ± 0.4 ^b^	655.8 ± 21.4 ^d^	437.7 ± 61.6 ^b^	1120.5 ± 5.3 ^c^
	2	278.2 ± 5.9 ^a^	9.4 ± 2.6 ^a^	5.2 ± 2.3 ^a^	2.1 ± 4.3 ^a^	30.8 ± 3.2 ^a^	21.9 ± 2.1 ^a^	36.0 ± 10.2 ^a^
	3	2732.5 ± 12.5 ^b^	89.9 ± 6.5 ^b^	98.6 ± 2.3 ^b^	55.8 ± 2.3 ^c^	501.6 ± 12.6 ^b^	564.5 ± 12.5 ^c^	985.6 ± 9.8 ^b^
	4	9972.9 ± 135.2 ^d^	201.6 ± 8.4 ^d^	125.6 ± 6.5 ^c^	69.8 ± 5.4 ^d^	611.6 ± 21.8 ^c^	800.7 ± 24.6 ^d^	2576.9 ± 66.5 ^d^
	5	34,787.3 ± 492.2 ^e^	243.1 ± 10.7 ^e^	209.6 ± 2.5 ^d^	70.8 ± 6.7 ^d^	855.3 ± 24.6 ^e^	1285.6 ± 56.1 ^e^	8542.3 ± 627.5 ^e^
‘Neb Jmel’	1	3559.9 ± 34.4 ^c^	108.5 ± 1.4 ^b^	102.6 ± 4.6 ^c^	17.0 ± 2.4 ^b^	355.4 ± 1.5 ^c^	331.2 ± 18.2 ^b^	629.9 ± 104.4 ^b^
	2	187.5 ± 10.3 ^a^	5.4 ± 2.1 ^a^	5.1 ± 1.8 ^a^	0.9 ± 3.4 ^a^	18.8 ± 3.1 ^a^	11.6 ± 3.7 ^a^	31.5 ± 10.6 ^a^
	3	1721.0 ± 33.7 ^b^	98.6 ± 2.3 ^b^	70.6 ± 7.5 ^b^	20.9 ± 2.6 ^b^	301.5 ± 10.2 ^b^	405.8 ± 14.5 ^c^	897.6 ± 9.9 ^c^
	4	5385.8 ± 34.9 ^d^	180.6 ± 10.3 ^c^	125.6 ± 3.8 ^c^	35.6 ± 3.2 ^c^	501.6 ± 11.6 ^d^	689.7 ± 8.9 ^d^	1890.1 ± 118.1 ^d^
	5	16,265.6 ± 306.2 ^e^	201.3 ± 26.2 ^c^	207.9 ± 8.9 ^d^	55.6 ± 1.2 ^d^	756.9 ± 14.6 ^e^	991.6 ± 6.7 ^e^	10,642.7 ± 333.2 ^e^
**Variety**	**E**	**Oleuropein**	**Quercetin**	**Gallic acid**	**Vanillic acid**	**Caffeic acid**	***p*** **-Coumaric acid**	**Chlorogenic acid**
‘Sayali’	1	15,781.7 ± 84.7 ^b^	1120.8 ± 22.7 ^c^	2.7 ± 0.2 ^a^	1.0 ± 0.1 ^a^	311.3 ± 9.8 ^b^	1.5 ± 0.0 ^a^	60.4 ± 10.3 ^a^
	2	849.1 ± 4.3 ^a^	156.0 ± 3.8 ^a^	nq	nq	18.6 ± 2.4 ^a^	nq	nq
	3	14,588.9 ± 71.6 ^b^	1079.2 ± 116.2 ^b^	3.0 ± 0.2 ^b^	1.3 ± 0.1 ^b^	365.4 ± 9.8 ^c^	1.9 ± 0.1 ^b^	74.8 ± 9.8 ^b^
	4	241,078.7 ± 105.0 ^c^	1194.8 ± 180.0 ^c^	5.0 ± 0.1 ^c^	2.6 ± 0.1 ^c^	453.5 ± 10.4 ^d^	3.0 ± 1.0 ^c^	80.5 ± 6.8 ^c^
	5	275,059.6 ± 69.8 ^d^	2980.6 ± 55.9 ^d^	6.4 ± 0.2 ^d^	3.3 ± 0.1 ^d^	532.9 ± 11.4 ^e^	3.8 ± 1.0 ^d^	117.7 ± 9.4 ^d^
‘Tkobri’	1	5406.2 ± 245.5 ^b^	1100.6 ± 55.4 ^b^	1.8 ± 0.1 ^a^	0.3 ± 0.1 ^a^	201.3 ± 11.3 ^b^	1.0 ± 0.1 ^a^	32.4 ± 10.2 ^a^
	2	230.3 ± 5.5 ^a^	75.0 ± 2.4 ^a^	nq	nq	11.1 ± 0.9 ^a^	nq	nq
	3	5287.9 ± 284.2 ^b^	1056.9 ± 48.9 ^b^	2.0 ± 0.1 ^b^	0.6 ± 0.1 ^b^	209.8 ± 10.4 ^b^	1.2 ± 0.1 ^b^	42.3 ± 9.6 ^a^
	4	243,177.8 ± 105.3 ^d^	1325.0 ± 139.3 ^c^	3.1 ± 0.1 ^c^	1.1 ± 0.1 ^c^	300.3 ± 9.9 ^c^	2.2 ± 0.1 ^c^	55.9 ± 7.8 ^b^
	5	203,808.5 ± 96.8 ^c^	1554.6 ± 23.5 ^d^	4.6 ± 0.1 ^d^	1.9 ± 0.1 ^d^	411.4 ± 9.9 ^d^	2.3 ± 0.1 ^c^	68.5 ± 8.6 ^c^
‘Neb Jmel’	1	6272.4 ± 204.1 ^b^	805.9 ± 55.7 ^c^	1.1 ± 0.1 ^a^	0.6 ± 0.1 ^a^	200.5 ± 11.5 ^b^	0.8 ± 0.0 ^a^	29.8 ± 9.7 ^a^
	2	253.6 ± 6.4 ^a^	50.3 ± 2.7 ^a^	nq	nq	12.0 ± 1.8 ^a^	nq	nq
	3	22,417.7 ± 434.4 ^c^	582.2 ± 40.6 ^b^	1.2 ± 0.1 ^a^	1.0 ± 0.1 ^bc^	227.8 ± 9.7 ^c^	1.0 ± 0.1 ^b^	25.9 ± 9.5 ^a^
	4	141,859.7 ± 105.6 ^d^	775.1 ± 96.3 ^c^	2.0 ± 0.1 ^b^	1.2 ± 0.1 ^c^	287.9 ± 8.6 ^d^	1.3 ± 0.1 ^c^	39.5 ± 10.3 ^b^
	5	186,837.0 ± 318.3 ^e^	1865.4 ± 20.8 ^d^	2.6 ± 0.1 ^c^	1.9 ± 0.1 ^d^	300.6 ± 10.6 ^e^	2.0 ± 0.2 ^d^	58.5 ± 11.2 ^c^

Results are expressed as mean ± standard deviation (SD) of the three sample replicates. Values represented by different small letters in the same column are statistically different (Tukey’s Honest Significant Difference (HSD) test, *p* < 0.05). 1: aqueous; 2: hexane; 3: diethyl ether; 4: ethyl acetate; 5: methanol. E: extraction; PB1: Procianidine B1; ns: not significant; nq: not quantified.

**Table 2:** Polyphenols concentration of aqueous OLE (mg 100 g^−1^) after in vitro gastrointestinal digestion.

**Variety**	**Phases**	**Hydroxtyrosol**	**Tyrosol**	**PB1**	**Epicatequin**	**Verbascoside**	**Quercetin-3-rutinoside**	**Luteolin-7-*O*-glucoside**
**‘Sayali’**	**Fresh**	9278.0 ± 14.4 ^f^	258.3 ± 6.2 ^e^	120.5 ± 7.9 ^f^	115.3 ± 2.5 ^f^	782.7 ± 12.9 ^e^	709.4 ± 3.8 ^d^	2007.9 ± 7.3 ^f^
	**O**	8521.7 ± 15.6 ^e^	224.6 ± 6.8 ^d^	114.6 ± 4.6 ^e^	105.9 ± 5.6 ^e^	755.9 ± 15.4 ^d^	700.4 ± 5.5 ^d^	1975.4 ± 9.8 ^e^
	**G**	2674.2 ± 21.4 ^d^	102.6 ± 5.4 ^c^	75.6 ± 5.5 ^d^	69.8 ± 3.4 ^d^	150.8 ± 9.6 ^c^	168.9 ± 6.8 ^c^	855.7 ± 7.4 ^d^
	**SI**	1650.9 ± 13.6 ^c^	92.4 ± 6.8 ^b^	60.7 ± 9.8 ^c^	52.9 ± 7.1 ^c^	125.4 ± 5.8 ^b^	115.7 ± 4.8 ^b^	655.8 ± 5.8 ^c^
	**LI**	1006.7 ± 9.8 ^a^	69.7 ± 7.9 ^a^	40.9 ± 4.7 ^a^	35.7 ± 2.5 ^a^	95.6 ± 6.7 ^a^	90.7 ± 9.8 ^a^	489.7 ± 10.2 ^a^
	**C-LI**	1250.6 ± 10.7 ^b^	75.9 ± 6.8 ^a^	55.9 ± 2.6 ^b^	40.8 ± 6.1 ^b^	110.4 ± 8.2 ^a^	101.7 ± 7.9 ^a^	521.7 ± 7.1 ^b^
**Variety**	**Phases**	**Oleuropein**	**Quercetin**	**Gallic acid**	**Vanillic acid**	**Caffeic acid**	***p*-Coumaric acid**	**Chlorogenic acid**
**‘Sayali’**	**Fresh**	15,781.7 ± 84.7 ^f^	1120.8 ± 22.7 ^f^	2.7 ± 0.2 ^f^	1.0 ± 0.1 ^b^	311.3 ± 9.8 ^e^	1.5 ± 0.0 ^c^	60.4 ± 10.3 ^d^
	**O**	12,358.7 ± 12.3 ^e^	1005.8 ± 5.9 ^e^	2.3 ± 0.1 ^e^	0.9 ± 0.1 ^b^	296.5 ± 8.5 ^d^	1.1 ± 0.1 ^b^	54.3 ± 8.7 ^c^
	**G**	5542.9 ± 20.4 ^d^	304.8 ± 6.4 ^d^	1.0 ± 0.0 ^d^	0.2 ± 0.1 ^a^	45.6 ± 5.4 ^c^	0.3 ± 0.1 ^a^	23.1 ± 8.6 ^b^
	**SI**	4987.6 ± 9.8 ^c^	255.9 ± 8.7 ^c^	0.9 ± 0.0 ^c^	nq	40.1 ± 2.6 ^b^	nq	18.3 ± 9.5 ^b^
	**LI**	2079.4 ± 11.7 ^a^	198.4 ± 11.6 ^a^	0.7 ± 0.0 ^a^	nq	34.2 ± 7.1 ^a^	nq	14.3 ± 4.8 ^a^
	**C-LI**	2555.7 ± 13.9 ^b^	225.7 ± 14.8 ^b^	1.0 ± 0.0 ^b^	nq	44.6 ± 2.7 ^c^	nq	19.9 ± 6.3 ^b^

Results are expressed as mean ± SD of the three sample replicates. Different small letters in the same columns indicate significant statistical differences (Tukey´s HSD test, *p* < 0.05) among treatments. Fresh: olive leaf extract; PB1: Procianidine B1; O: oral phase; G: gastric phase; SI: small intestine phase; LI: large intestine phase; C-LI: control large intestine phase; ns: not significant; nq: not quantified.

**Table 3:** Phenolic composition (mg 100 g^−1^) of Californian-style table olives of ‘Hojiblanca’ variety with and without the addition of aqueous OLE of ‘Sayali’ variety.

‘Hojiblanca’		
Phenolic Profile (mg 100 g^−1^)	Oxidized Black Olive (T)	(T) + OLE (1:10)
Hydroxytyrosol	464.3 ± 10.3 ^a^	2171.5 ± 30.3 ^b^
Tyrosol	25.4 ± 4.5 ^a^	82.1 ± 8.9 ^b^
PB1	18.3 ± 4.3 ^a^	55.5 ± 4.3 ^b^
Epicatechin	2.7 ± 2.2 ^a^	42.7 ± 9.1 ^b^
Verbascoside	3.9 ± 0.2 ^a^	284.9 ± 10.4 ^b^
Quercetin-3-rutinoside	2.3 ± 1.2 ^a^	300.1 ± 12.0 ^b^
Luteolin-7-*O*-glucoside	2.4 ± 1.3 ^a^	570.0 ± 45.6 ^b^
Oleuropein	110.2 ± 14.6 ^a^	3460.9 ± 75.6 ^b^
Quercetin	nq	295.7 ± 32.5
Vanillic acid	2.9 ± 0.3 ^ns^	2.9 ± 0.5 ^ns^
Caffeic acid	nq	124.9 ± 10.3
*p*-Coumaric	8.4 ± 0.7 ^ns^	6.9 ± 1.3 ^ns^
Chlorogenic acid	nq	25.2 ± 3.6
Σ phenols	640.8 ± 6.8 ^a^	7423.3 ± 15.6 ^b^

Results are expressed as mean ± SD of the three sample replicates. Different small letters in the same row indicate significant statistical differences (Tukey´s HSD test, *p* < 0.05) among treatments. PB1: Procianidine B1; ns: not significant; nq: not quantified.

**Table 4:** Polyphenol compounds (mg 100 g^−1^) of Californian-style table olives after addition of aqueous OLE and submitted to in vitro gastrointestinal digestion.

‘Hojiblanca’					
Phenolic Profile (mg 100 g^−1^)	O	G	SI	LI	C-LI
Hydroxytyrosol	1843.4 ± 45.6 ^e^	803.1 ± 98.7 ^d^	410.6 ± 56.5 ^c^	256.4 ± 20.7 ^a^	311.2 ± 30.7 ^b^
Tyrosol	70.3 ± 1.4 ^d^	30.1 ± 2.1 ^c^	13.4 ± 2.2 ^b^	8.9 ± 2.4 ^a^	10.7 ± 1.1 ^a^
PB1	50.4 ± 3.5 ^d^	20.2 ± 1.5 ^c^	10.1 ± 2.2 ^b^	6.7 ± 2.3 ^a^	8.4 ± 2.1 ^a^
Epicatechin	38.8 ± 4.3 ^d^	16.1 ± 1.1 ^c^	7.2 ± 2.0 ^b^	4.8 ± 1.1 ^a^	5.8 ± 1.4 ^a^
Verbascoside	260.1 ± 21.4 ^d^	104.0 ± 2.1 ^c^	52.0 ± 2.5 ^b^	34.7 ± 2.3 ^a^	40.6 ± 2.2 ^a^
Quercetin-3-rutinoside	272.8 ± 13.3 ^d^	109.1 ± 3.0 ^c^	54.6 ± 3.4 ^b^	36.4 ± 3.1 ^a^	43.7 ± 3.3 ^a^
Luteolin-7-*O*-glucoside	500.3 ± 25.5 ^e^	200.1 ± 30.8 ^d^	100.1 ± 67.1 ^c^	66.7 ± 7.8 ^a^	80.0 ± 7.2 ^b^
Oleuropein	3146.3 ± 89.5 ^e^	1258.5 ± 90.1 ^d^	629.3 ± 95.2 ^c^	419.5 ± 25.2 ^a^	497.5 ± 45.2 ^b^
Quercetin	268.8 ± 14.5 ^d^	107.5 ± 5.1 ^c^	53.8 ± 6.8 ^b^	35.8 ± 5.8 ^a^	40.0 ± 5.1 ^a^
Vanillic acid	2.7 ± 0.3 ^b^	1.1 ± 0.2 ^a^	nq	nq	nq
Caffeic acid	113.5 ± 12.4 ^c^	45.4 ± 13.2 ^b^	22.7 ± 6.4 ^a^	15.1 ± 5.2 ^a^	18.2 ± 4.6 ^a^
*p*-Coumaric	6.2 ± 2.4 ^b^	2.5 ± 2.1 ^a^	nq	nq	nq
Chlorogenic acid	22.9 ± 3.3 ^c^	9.2 ± 1.3 ^b^	4.6 ± 1.4 ^a^	3.1 ± 0.6 ^a^	3.7 ± 0.7 ^a^
Σ phenols	6596.7 ± 18.6 ^e^	2706.9 ± 31.6 ^d^	1358.3 ± 37.2 ^c^	888.2 ± 41.1 ^a^	1059.7 ± 40.2 ^b^

Values represented by different small letters in the same column are statistically different (Tukey´s HSD test, *p* < 0.05). PB1: Procianidine B1; O: oral phase; G: gastric phase; SI: small intestine phase; LI: large intestine phase; C-LI: control large intestine phase; ns: not significant; nq: not quantified.

## Data Availability

The authors confirm that the data supporting the findings of this study are available within the article and that the raw data supporting the findings are available from the corresponding author, upon reasonable request.

## Sample Availability

Samples of the compounds are available from the authors.
